# Literature and License

**Published:** 1921-08-06

**Authors:** 


### LITERATURE AND LICENSE.

**(From a Correspondent.)**

It is a commonplace to say that we live in an age of change. The remark is made by many with an unction which demonstrates chiefly their ignorance that in all times it has been possible to make the same comment. Even Lucretius noticed the fact that everything is in a state of flux! Yet there are some people who—like the courtiers of Canute—believe that the tide can be stayed, and, consequently, they either implore or demand that someone should oppose it, or they struggle with all their puny strength to check the waves of progress. We may sympathise with their desires—even commend them—if we are convinced that what they oppose is evil; but often enough it is difficult to say whether the thing is evil or not. It may only be justified by its results. Such, for example, we may consider inoculation or vaccination against small-pox, or the injection of nasty and unpleasant anti-toxins to ward off worse disorders. But to combat suggestions because the immediate effects are unpleasant is to take a short-sighted view of life, and thus to hamper even the small amount of progress that is possible to us. As a race we are cautious; and it is more pleasant to designate our attitude as one of philosophic doubt rather than to call it obstinacy or impermeability to new ideas!

Indeed, it is not even a question of new ideas. Our knowledge of history is so limited that, often enough, we resent the novelty of something that was antique before we were in existence as a nation. In the matter of religion we are ceasing to be shocked when the sceptic insists that, for example, there is some doubt as to whether Jonah really took lodgings in the whale's interior. When, however, the discussion turns upon questions pertaining to sex it becomes incumbent upon most of us to adopt the Oh-no-we-never-mention-it attitude. It seems as if liberties were being taken with our so well-known innocence in thought and word! And yet how many of us are there who can, when we read the confessions of such men as Pepys, Rousseau, or Cellini, say that we are—from the point of view now under discussion—better or more chaste in thought than they? Perhaps even our actions might bear the light of day less favourably.

Not that it is considered desirable that every form of censorship should be abrogated—though even then we should work out our own literary salvation—but it ought not to be difficult to devise some system with greater breadth and catholicity than the present one. Consider the state of affairs which makes it possible to ban such books as Havelock Ellis's "Psychology of Sex" and yet permits the publication of many other books of merely quasi-scientific interest! Fortunately there are signs of enlightenment. It is now possible to discuss freely and frankly the problems connected with the prevention or treatment of venereal disease—though even in this respect our national characteristic is to be noted. We do realise the need for treatment; but suggestions as to prophylaxis are not allowed to be entirely untinged by original sin! But what a great difference there is between the time when Mrs. Besant and Charles Bradlaugh were attacked for their advocacy of Malthusian principles and the present day, when the books of Dr. Marie Stopes are allowed free circulation (except, is it not, in that home of propriety the United States of America—some of the conditions there existing having been made clear in the pages of "Susan Lenox"?).

I am moved to these remarks by the fact that I have recently read "The Autobiography of a Child." It will be remembered that certain of the City Fathers sat down to a consideration of this volume, and thereafter decreed that it should be burnt by the public executioner—or whoever the worthy is who immolates erring volumes of an immoral tendency. It is now, as the booksellers remark, scarce; and a copy can only be obtained surreptitiously and for many shekels. I hasten to add that it was lent to me. And now I wonder what all the pother was about. There is a man of no mean degree of culture who has the courage to sit down and write an account of his childhood, not glozing over what is usually either omitted altogether or else ascribed to someone else, but withal not offensively advertising the sexual portion of his history. Indeed, the part dealing with his sexual evolution is not of such predominance as might be expected from all the accounts we have had; as a matter of fact, most of the paragraphs to which exception has been taken are quoted from other books which still remain on sale. Why not, then, in the words of one who apparently had little use for hypocrites, "Sack the lot!" It would be a logical—if stupid—conclusion. And that is not difficult for the dwellers in "Stupidity Street."

Apart from the sexual portion of the book there is much excellent character-drawing; while the description of the various places in which he lived both at home and abroad is done by one who has observed with much care, and who has also been endowed with a retentive memory. Considered from any point of view, it is difficult to understand how such a book can be classified with prurient literature. To use a well-worn phrase, it is indeed a human document; and if it must be veiled from sight of the immature and uncultivated—who don't read such books unless they are advertised by the keepers of the public morals!—then let it be issued to the limited circle of those whose morals are either safe or incapable of being made worse. Far be it from me to specify who shall be included therein. Seriously, however, it is time that we had done with this grandmotherly coddling of our intellect. Or should we prescribe for our arbiters of morals a course of Rabelais, Casanova, Boccaccio, and so on, before they are allowed to enter upon their duties? The works of these writers and of others with a like catholicity of taste—and breadth of expression—can be obtained easily from any competent bookseller!

